# Sex-Based Differences in Pulmonary Function and Cardiopulmonary Response 30 Months Post-COVID-19: A Brazilian Multicentric Study

**DOI:** 10.3390/ijerph21101293

**Published:** 2024-09-27

**Authors:** Cássia da Luz Goulart, Guilherme Peixoto Tinoco Arêas, Mauricio Milani, Fernanda Facioli dos Reis Borges, Juliana Ribeiro Magalhães, Guilherme Dionir Back, Audrey Borghi-Silva, Luciano Fonseca Lemos Oliveira, André Ribeiro de Paula, Carolina Coimbra Marinho, Déborah Pereira Prado, Celso Nascimento de Almeida, Cristiane Maria Carvalho Costa Dias, Vinícius Afonso Gomes, Luiz Eduardo Fonteles Ritt, Leandro Tolfo Franzoni, Ricardo Stein, Mansueto Gomes Neto, Gerson Cipriano Junior, Fernando Almeida-Val

**Affiliations:** 1 Physiological Science Department, Universidade Federal do Amazonas, Manaus 69067-005, Brazil; luz.cassia@hotmail.com (C.d.L.G.); guilhermepta@ufam.edu.br (G.P.T.A.); j_magal@hotmail.com (J.R.M.); 2 Fundação de Medicina Tropical Dr. Heitor Vieira Dourado (FMT-HVD), Av. Pedro Teixeira, N25-Bairro Dom Pedro, Manaus 69040-000, Brazil; 3 Research Group on Cardiopulmonary Rehabilitation (GPRC), University of Brasilia (UnB), Brasilia 70910-900, Brazil; milani@medicinadoexercicio.com (M.M.); ciprianeft@gmail.com (G.C.J.); 4 Rehabilitation Research Center, Faculty of Rehabilitation Sciences, Hasselt University, 3590 Diepenbeek, Belgium; 5 Cardiopulmonary Physical Therapy Laboratory, Federal University of São Carlos, São Carlos 13565-905, Brazil; guilhermeback4@gmail.com (G.D.B.); audrey@ufscar.br (A.B.-S.); 6 Department of Internal Medicine, Federal University of Minas Gerais (UFMG), Belo Horizonte 30130-100, Brazil; oliveiralfl@hotmail.com (L.F.L.O.); andreribeirofisioterapia@gmail.com (A.R.d.P.); carolina.marinho@ebserh.gov.br (C.C.M.); dpprado@gmail.com (D.P.P.); 7 Bahiana Cardiorespiratory Physiotherapy Research Group, Bahiana School of Medicine and Public Health (EBMSP), Salvador 40290-000, Brazil; celsoalmeida.pos@bahiana.edu.br (C.N.d.A.); cmccdias@bahiana.edu.br (C.M.C.C.D.); vinifisioterapia@yahoo.com.br (V.A.G.); luizritt@bahiana.edu.br (L.E.F.R.); 8 Department of Clinical Medicine, Federal University of Rio Grande do Sul, Porto Alegre 90010-150, Brazil; lfranzoni@hcpa.edu.br (L.T.F.); rstein@cardiol.br (R.S.); 9 Physiotherapy Research Group, Federal University of Bahia, Salvador 40170-110, Brazil; mansueto.neto@ufba.br

**Keywords:** COVID-19, long-COVID, cardiopulmonary health, exercise test, cardiorespiratory fitness

## Abstract

**Aim**: (I) to verify if there are sex differences in respiratory function, respiratory muscle strength, and effort limitation in individuals recovered from severe acute COVID-19 30 months after the initial infection, and (II) to evaluate the influence of length of stay on cardiorespiratory capacity among men and women. **Methods**: Cross-sectional observational multicentric study with participants from five Brazilian states (São Paulo, Amazonas, Minas Gerais, Bahia, and Brasília). We assessed lung function and respiratory muscle strength by maximum inspiratory pressure (MIP), maximum expiratory pressure (MEP), and cardiorespiratory fitness by cardiopulmonary exercise test (CPET). **Results**: 86 individuals were stratified by sex (48 women and 38 men). Females had significantly longer hospitalization for acute COVID-19 (*p* < 0.05) and showed a marked reduction in MIP (cmH_2_O and % predicted). Regarding the CPET, women presented lower $\dot{V}$O_2_% predicted, O_2_ pulse, and oxygen uptake efficiency slope (OUES, % predicted) (*p* < 0.05). In addition, women also had greater abnormal combinations between RER < 1.10, OUES < 80% predicted, VE/VVM < 15% [3 (6.2%)] and $\dot{V}$O_2_% predicted < 80%, $\dot{V}$E/$\dot{V}$CO_2_ slope and $\dot{V}$O_2_/workload < 8.4 mL/min/W [8 (17%)]. The regression analysis showed a significant influence of age, length of hospitalization (< and >10 days), and FEV_1_/FVC (%) on the $\dot{V}$O_2_ peak (mL·kg^−1^·min^−1^). Secondarily, we found that women hospitalized for more than 10 days had worse O_2_ pulse (*p* = 0.03), OUES % predicted (*p* < 0.001), and worse $\dot{V}$O_2_% predicted (*p* < 0.009). **Conclusion**: Women exhibited more pronounced impairments in several key indicators of cardiopulmonary function 30 months post-infection.

## 1. Introduction

Long COVID is a multisystem disorder associated with over 200 different symptoms [1], affecting about 150 million people worldwide who experience at least one persistent symptom [2]. The quality of life and physical and functional capacity are profoundly affected [3,4], preventing individuals from engaging in leisure and work activities [5,6]. Sex differences in exercise outcomes are well documented, influencing performance assessment, susceptibility to fatigue, and risk of injury [7]. Studies have shown a different incidence of long COVID according to sex, hampering prompt comprehension of such cardiopulmonary function and recovery patterns [8,9].

In the context of COVID-19, men and women may present distinct recovery patterns and responses to exercise. For instance, hormones, body composition, and ventilatory capacity have been hypothesized to influence the response to exercise after recovery from COVID-19 [10,11]. Furthermore, evidence on how exercise response and physical recovery from the disease differ between sexes is scarce and merits further comprehension [8,9].

It remains uncertain whether women who had severe acute COVID-19 have worse cardiorespiratory outcomes after years of the acute disease episode. Therefore, this study aims to verify if there are sex differences in respiratory function, respiratory muscle strength, and effort limitation in individuals who recovered from severe acute COVID-19 30 months after the initial infection. We hypothesize that women with long COVID have worse physical and functional outcomes.

## 2. Methods

### 2.1. Study Design and Ethical Considerations

This was a Brazilian multicentric cross-sectional observational study, which included participants from Manaus, in the western Brazilian Amazon (Fundação de Medicina Tropical Doutor Heitor Vieira Dorado, FMT-HVD and Universidade Federal do Amazonas, UFAM) form Brasília, in the central-west region (Universidade de Brasília, UnB); from São Carlos (Universidade Federal de São Carlos, UFSCar) and Belo Horizonte (Universidade Federal de Minas Gerais, UFMG) at the Southeast Brazilian region, and in Bahia (Escola Bahiana de Medicina e Saúde Pública, EBMSP), northeast region.

This study followed the Strengthening the Reporting of Observational Studies in Epidemiology (STROBE) [12] statement. The study was approved by the human research ethics committee of the Federal University of Amazonas, register number #64127022.5.0000.5020. All procedures were carried out following good clinical and laboratory practices and the precepts of the Declaration of Helsinki and the Committee for International Harmonization for Human Research.

### 2.2. Population, Study Groups, and Eligibility Criteria

Participants included men and women over 18 years old diagnosed with severe COVID-19 during the acute phase according to the World Health Organization (WHO) [13] criteria (Clinical signs of pneumonia are present, such as fever, cough, and difficulty breathing; Respiratory rate is ≥30 breaths per minute in adults; Oxygen saturation is <90% on room air and Imaging shows significant lung involvement). Eligibility required results from a positive polymerase chain reaction (RT-PCR) test for SARS-CoV-2 at the time of hospitalization for severe COVID-19. Non-inclusion criteria consisted of pulmonary rehabilitation six months before study inclusion, peripheral saturation (SpO_2_) at rest lower than 88% at the time of CPET assessment, individuals with pacemaker or implantable defibrillator, presence of a chronic-degenerative, osteoarticular or muscle dysfunction that would prevent the proposed assessments from being carried out, decompensated oncological, metabolic or renal disease, uncontrolled systemic arterial hypertension, alcoholism, current or ex-smokers, history of illicit drug abuse, pregnancy or claustrophobia due to the use of a mask when performing the exercise test.

### 2.3. Recruitment Strategy and Participant Inclusion

Informal posters were disseminated via social media and placed in high-circulation localities throughout the inclusion cities to reach individuals with the eligibility criteria. In addition, contact was made with participants from other previous studies involving COVID-19. Once the eligibility criteria were met, the participant’s assessment was scheduled. For all participants included in the study, the following procedures were performed: informed consent, assessment of medical history, clinical evaluation, spirometry, manovacuometry, and cardiopulmonary exercise testing.

### 2.4. Clinical Assessments

An interview was conducted to collect sociodemographic, epidemiological, and medical history. Data collection included COVID-19 infection history, diagnosis dates, hospital admission and discharge, the severity of infection, previous illness, comorbidities, blood pressure, heart rate, body mass index (BMI), and fatigue assessment using the Portuguese Modified Medical Research Council (mMRC) scale. After this interview, the physical evaluations were performed.

### 2.5. Lung Function

Spirometry was performed according to the American Thoracic Society (ATS) guidelines [14]. The following variables were assessed: forced expiratory volume at the first second (FEV_1_), forced vital capacity (FVC), and FEV_1_/FVC. We used the reference values described for the Brazilian population [15]. During the tests, specific disposable antibacterial/antiviral filters were used to prevent contamination of the device and participants.

### 2.6. Respiratory Muscle Strength

Manovacuometry was performed to obtain the maximum inspiratory (MIP) and maximum expiratory pressures (MEP). The tests followed the recommendations of the ATS/European Respiratory Society (ERS) [16]. The predicted value of MIP was according to Pessoa [17], and the criteria for inspiratory muscle weakness were determined by Rodrigues [18].

### 2.7. Cardiopulmonary Exercise Testing

Ramp-incremental exercise tests were performed on an electronically braked cycle ergometer on a maximum ramp-incremental exercise test with an individually adjusted load (5–15 W/min) at 60 rpm, preceded by unloaded pedaling at 0 W for 2 min. Oxygen uptake and carbon dioxide production ($\dot{V}$O_2_, and $\dot{V}$CO_2_, mL/min) and minute ventilation (VE, L/min) were measured by a breath-by-breath metabolic cart with a 12-lead electrocardiogram for heart rate (HR, bpm). The oxygen uptake efficiency slope (OUES) was calculated, and the gas exchange threshold was estimated by visually inspecting the inflection point on the $\dot{V}$CO_2_ versus $\dot{V}$O_2_ relationship (i.e., V-slope method) [19]. The criteria to identify maximal effort were the highest O_2_ uptake obtained during exercise (30-s average) and a peak RER of 1.10 or above [20,21,22]. $\dot{V}$O_2_ [23] and OUES [24] predicted were calculated based on reference values for the Brazilian population.

The $\dot{V}$O_2_/workload peak was measured from the $\dot{V}$O_2_ peak (L/kg/min)/workload peak. Values <8.4 mL/min/W were considered abnormal. Patients were divided into subgroups based on normal or abnormal $\dot{V}$O_2_/workload relationship [25]. Ventilatory efficiency was estimated from the ventilatory equivalent for carbon dioxide ($\dot{V}$E/$\dot{V}$CO_2_) slope. Values >34 were considered abnormal [26]. The indirect maximal voluntary ventilation (MVV), calculated as FEV1 × 40, was used to estimate the breathing reserve (%) as follows: [MVV − minute ventilation [VE])/MVV) × 100. VE close to MVV indicates a low breathing reserve, and a breathing reserve <15% was considered abnormal [26].

### 2.8. Statistical Analysis

Data were expressed as mean ± standard deviation (SD) or median with interquartile range (IQR) depending on data distribution, which was tested by the Shapiro-Wilk and Levene tests for homogeneity. Differences between groups were presented in percentage and 95% confidence interval. The unpaired *t*-student test was used for means, and the Mann-Whitney was used for medians to analyze the difference between groups. The X^2^ test tested the distribution of categorical variables among groups. We analyzed the difference between the sexes and the length of hospitalization in the acute phase of COVID-19 through an ANOVA two-way test (between factors) with a post hoc Bonferroni test. The multiple method was used for regression analysis, evaluating β and 95% interval confidence. The *p*-value < 0.05 was accepted as statistically significant. To evaluate the effect size, Cohen’s D value was used for normal (parametric) data and the order biserial correlation for non-parametric data. Data were processed in IBM-SPSS 24.0 for MAC and GraphPad (Prism 7.0, USA).

## 3. Results

A total of 86 individuals (48 females and 38 males) were included in this study. Firstly, we analyzed the past medical history of the study participants, which revealed that women experienced significantly longer hospital stays compared to men (*p* < 0.05). No significant disparities were observed between the two groups regarding age, weight, body mass index (BMI), Modified Medical Research Council (mMRC) dyspnea scale scores, or the distribution of comorbidities (Table 1).

The pulmonary function test and the assessment of the respiratory muscle strength revealed a significantly lower MIP as a percentage of the predicted values, with large effect sizes (*p* < 0.05) in women (Table 2). No significant differences between men and women were observed in pulmonary function or MEP.

Regarding the CPET assessments, women exhibited a reduced peak work rate (WR) (*p* = 0.01, Cohen’s D 0.70), a reduced $\dot{V}$O_2_ as a percentage of the predicted value (*p* = 0.01, Cohen’s D 0.58), a reduced oxygen pulse (*p* = 0.02, Cohen’s D 0.29), and a reduced OUES as a percentage of the predicted value (*p* = 0.05, Cohen’s D 0.45) compared to men (Table 3), with similar respiratory exchange ratio (RER) between groups.

Regression analysis indicated a significant influence of age, length of hospital stay (< or >10 days), and FEV_1_/FVC (%) on the peak $\dot{V}$O_2_ (mL·kg^−1^·min^−1^), collectively explaining 40% of the variance in peak $\dot{V}$O_2_ (kg/L/min) (Table 4). 

**Figure 1:**
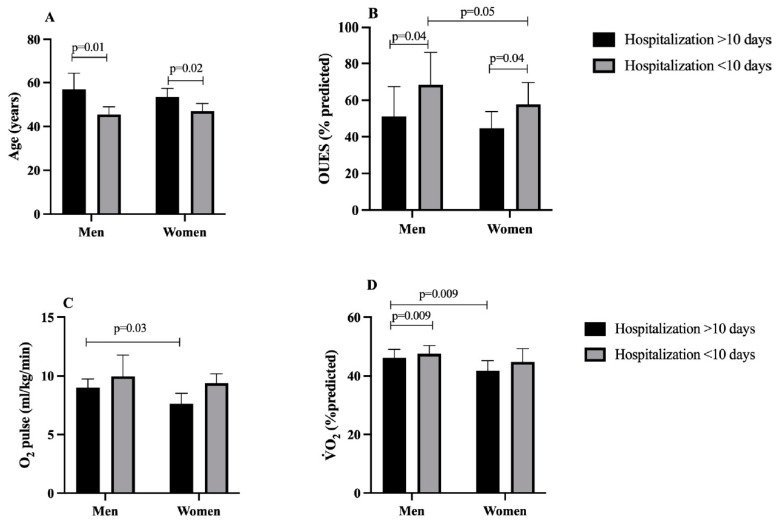
Stratification of patients by sex (men and women) and days of hospitalization (more and less than 10 days) in the acute phase of COVID-19 to evaluate differences in (**A**) age, (**B**) OUES (% predicted), (**C**) O_2_ pulse and (**D**) $\dot{V}$O_2_ (% predicted). Notes: oxygen uptake efficiency slope (OUES); ANOVA two-way.

We further stratified participants based on length of hospitalization (< or >10 days) to examine the impact of the length of stay between groups (men and women) (Figure 1). We found that for both groups, those with >10 days were older (*p* = 0.01 for men and 0.02 for women) (Figure 1A). Additionally, hospital stays longer than 10 days resulted in worse OUES (% predicted) for both men and women (*p* = 0.04) and for women hospitalized for less than 10 days (Figure 1B) (*p* < 0.05). Moreover, women hospitalized for more than 10 days had worse O_2_ pulse (Figure 1C) (*p* = 0.03), and $\dot{V}$O_2_% predicted (Figure 1D) (*p* < 0.009). Similarly, men with hospital stay longer than 10 days had worse $\dot{V}$O_2_% predicted compared to men with shorter stays (Figure 1D) (*p* < 0.009).

**Figure 2:**
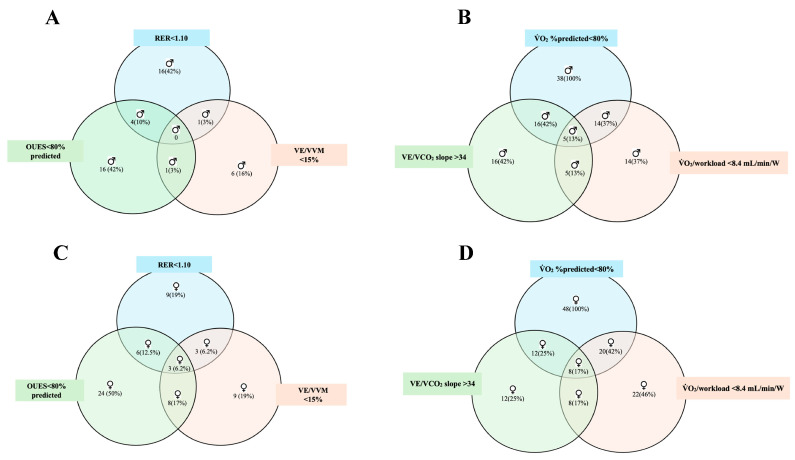
Venn diagram depicting the overall differences between females (**C**,**D**) and males (**A**,**B**) with an abnormal RER < 1.10, OUES < 80% predicted, VE/VVM < 15%, $\dot{V}$O_2_% predicted < 80%, $\dot{V}$E/$\dot{V}$CO_2_ slope, and $\dot{V}$O_2_/workload < 8.4 mL/min/W. The figure illustrates the N^o^ of patients (%) within each area. Chi-square: RER < 1.10 *p* = 0.34; OUES < 80% predicted *p* = 0.05; VE/VVM < 15% *p* = 0.87; $\dot{V}$O_2_% predicted < 80% NA; $\dot{V}$E/$\dot{V}$CO_2_ slope *p* = 0.06; $\dot{V}$O_2_/worload < 8.4 *p* = 0.10.

The overlap of patients with an abnormal RER < 1.10, OUES < 80% predicted, VE/VVM < 15%, $\dot{V}$O_2_% predicted < 80%, $\dot{V}$E/$\dot{V}$CO_2_ slope and $\dot{V}$O_2_/workload < 8.4 mL/min/W is illustrated in Figure 2. We found that women had greater ventilatory dysfunction by OUES < 80% predicted [24 (50%) *p* = 0.05] when compared to men; in addition, women also had greater abnormal combinations between RER < 1.10, OUES < 80% predicted, VE/VVM < 15% [3 (6.2%)] and $\dot{V}$O_2_% predicted < 80%, $\dot{V}$E/$\dot{V}$CO_2_ slope and $\dot{V}$O_2_/workload < 8.4 mL/min/W [8 (17%)].

## 4. Discussion

This is the first multicentric study to assess sex-based differences in pulmonary function, respiratory muscle strength, and cardiopulmonary response during exercise testing among patients who had had severe acute COVID-19 before the vaccination roll-out and recovered from the disease. The findings indicate significant disparities, with women exhibiting more pronounced impairments in several key indicators of cardiopulmonary function 30 months post-acute infection. These differences underscore the necessity for sex-specific follow-up and treatment strategies in the management of long COVID-19, aiming to address the unique recovery challenges faced by women.

Regarding respiratory muscle strength, a significant reduction in MIP (cmH_2_O and % predicted) observed in women suggests greater respiratory muscle weakness, adversely affecting long-term functional capacity and quality of life. This finding aligns with previous research indicating that long-term respiratory complications in COVID-19 survivors may persist well beyond the acute phase of the infection. The absence of differences observed in MEP might be attributed to the muscle-specific impact of COVID-19, or it may reflect the greater functional significance and vulnerability of inspiratory muscles compared to expiratory muscles. This suggests that the inspiratory muscles, which play a crucial role in breathing and overall respiratory function, are more adversely affected in the long-term aftermath of severe COVID-19. These persistent impairments highlight the critical need for targeted rehabilitation strategies to address and mitigate the long-term consequences of COVID-19 on respiratory muscle function, particularly in women [27].

Specifically, women demonstrated a reduced WR, reduced $\dot{V}$O_2_% predicted, reduced O_2_ pulse, and OUES% predicted when compared to men. These results indicate lower exercise capacity, reflecting less effective recovery of the cardiopulmonary system in women and potential long-term impacts on muscle metabolism and cardiac output, which have been observed in long-term COVID-19. A recent meta-analysis, which reviewed 38 studies encompassing CPET data on 2160 individuals, showed a significant reduction (4.9 mL·kg^−1^·min^−1^) in cardiorespiratory fitness among individuals with long COVID compared to those without symptoms for more than three months post-infection [28]. The underlying mechanisms reported for exercise intolerance are wide-ranging and include deconditioning, dysfunctional breathing, chronotropic incompetence, and abnormal peripheral oxygen extraction and utilization. 

Regression analysis identified a significant influence of age, duration of hospitalization during the acute phase (in days), and the FEV_1_/FVC (%) ratio on the peak $\dot{V}$O_2_ (kg/L/min) response. These factors collectively explained 40% of the variation in peak $\dot{V}$O_2_ (kg/L/min), suggesting that biological and clinical characteristics play a crucial role in cardiopulmonary recovery. The duration of hospitalization emerged as a particularly important factor, with women hospitalized for less than 10 days showing worse OUES (% predicted) compared to men. Current evidence shows a large discrepancy in findings, partially attributed to variability in methodology and interpretation between studies.

The observed differences in O_2_ pulse, an indicator of stroke volume and arterial oxygen content per heartbeat, further underscore potential sex-specific cardiovascular impairments in post-COVID-19 patients. COVID-19 can lead to varying degrees of cardiovascular dysfunction, which may evolve into chronic conditions more readily in women due to differences in cardiac physiology and recovery trajectories between sexes. The differences in O_2_ pulse between healthy controls and COVID-19 survivors might reflect the disease’s impact on cardiorespiratory fitness.

CPET results depended on the initial disease severity about eight months after COVID-19 onset, with hospitalized participants achieving significantly lower O_2_ pulse than non-hospitalized patients. However, in our study, we observed that even 30 months after acute COVID-19, patients still exhibited reduced O_2_ pulse, especially women who remained hospitalized for more than 10 days. This finding highlights the enduring impact of severe COVID-19 on cardiovascular health, emphasizing the need for long-term monitoring and tailored rehabilitation strategies to address these persistent impairments.

Similarly to the O_2_ pulse, OUES values represent an individual’s cardiorespiratory reserve and indicate how effectively oxygen is extracted and utilized by the body [29]. The lower OUES observed in women implies a less efficient ventilatory response during exercise, suggesting possible ongoing challenges in pulmonary function and reduced gas exchange efficiency compared to men. This inefficiency could indicate a prolonged recovery phase or possibly permanent lung tissue and function changes.

These findings underscore the complexity of Long COVID-19 recovery and the importance of considering specific patient factors when developing rehabilitation strategies. Women may require targeted interventions to improve respiratory muscle function and exercise capacity. Importantly, all participants had severe acute COVID-19 before the vaccine roll-out and from infection from early SARS-CoV-2 variants, which have implications for long COVID phenotype.

Our study has several limitations. The study’s cross-sectional and observational nature may have limited our ability to monitor the progression of patients over various time periods. Controlling for all variables in a cross-sectional study presents a significant challenge. The initial design of our study focused solely on evaluating severe patients in the acute phase of COVID-19. Although we recognize the importance of comparing patients across all severity levels, our study is innovative in presenting the results of lung function and exercise capacity in severe patients 30 months post-hospitalization for COVID-19 and from a varied geographical origin in such an extensive country as Brazil. Future research should delve deeper into the underlying mechanisms behind these observed differences and assess the efficacy of targeted interventions to enhance long-term health outcomes in this population.

## 5. Conclusions

Our multicenter study revealed significant sex-based differences in respiratory muscle strength and cardiopulmonary response during exercise testing among patients who have recovered from severe acute COVID-19. Women exhibited more pronounced impairments in several key indicators of cardiopulmonary function 30 months post-infection. These differences highlight the need for differentiated and personalized approaches in the follow-up and treatment of long-term COVID-19 patients, considering the specificities of each sex. Moreover, these findings underscore the importance of tailored rehabilitation strategies to address these sex-specific disparities, focusing on improving respiratory muscle strength, cardiovascular health, and overall exercise capacity, particularly for women.

## Figures and Tables

**Table 1:** Baseline characteristics of groups.

Variables	All Patients (*n* = 86)	Women (*n* = 48)	Men (*n* = 38)	*p* Value
Age (years)	50 ± 10	50 ± 10	49 ± 11	0.59
Weight (kg)	81 ± 14	81 ± 15	80 ± 13	0.66
BMI (kg/m^2^)	29 ± 5	29 ± 6	28 ± 5	0.35
mMRC	1.4 ± 0.6	1.3 ± 0.5	1.5 ± 0.6	0.29
Hospital stays (days)	19 ± 4	25 ± 3	12 ± 2	0.02
Assessment time after hospital discharge (months)	30 ± 5	31 ± 2	29 ± 4	0.12
Comorbidities, *n* (%)				
Hypertension	33 (38)	20 (42)	13 (34)	0.22
Diabetes	50 (58)	14 (29)	18 (47)	0.17

Notes: Student’s *t*-test; Body mass index (BMI); Modified Medical Research Council (MRC).

**Table 2:** Function and respiratory muscle strength of the groups.

Variables	All Patients (*n* = 86)	Women (*n* = 48)	Men (*n* = 38)	Cohen’s D	Mean Difference, (95% CI), *p* Value
FEV_1_ (L)	3.0 ± 0.6	3.2 ± 0.8	2.6 ± 0.1	0.16	−0.2 (−0.6 to 0.10), 0.53
FEV_1_ (% predicted)	82 ± 18	79 ± 14	85 ± 21	0.32	−5.7 (−17.0 to 5.5), 0.30
FVC (L)	3.1 ± 0.8	3.0 ± 0.7	3.1 ± 0.9	0.11	−0.1 (−0.6 to 0.28), 0.62
FVC (% predicted)	80 ± 15	78 ± 8	81 ± 5	0.09	−1.4 (−11.0 to 8.1), 0.76
FEV_1_/FVC (%)	80 ± 6	78 ± 7	81 ± 5	0.36	−0.04 (−0.12 to 0.03), 0.18
MIP (cmH_2_O)	80 ± 31	67 ± 28	91 ± 29	0.81	−23.8 (−45.4 to −2.2), 0.03
MIP (% predicted)	103 ± 39	87 ± 9	115 ± 9	0.76	−10.8 (−11.4 to 7.5), 0.04
MEP (cmH_2_O)	104 ± 30	95 ± 30	112 ± 29	0.59	−16.9 (−39.0 to 5.0), 0.12

Notes: Student’s *t*-test: Forced expiratory volume at the first minute (FEV_1_), forced vital capacity (FVC), maximum inspiratory pressure (MIP), and maximum expiratory pressure (MEP).

**Table 3:** Characteristics of the groups during the CPET.

	All Patients (*n* = 86)	Women (*n* = 48)	Men (*n* = 38)	Cohen’s D	Mean Difference, (95% CI), *p* Value
**Rest CPET**					
HR (bpm)	85 ± 15	84 ± 14	86 ± 13	0.15	−2.1 (−8.1 to 3.9), 0.42
SBP (mmHg)	123 ± 15	123 ± 17	124 ± 13	0.10	−1.6 (−11.3 to 8.0), 0.73
DBP (mmHg)	81 ± 8	81 ± 9	81 ± 7	0.01	0.10 (−0.5 to 5.2), 0.96
**Peak CPET**					
HR (bpm)	138 ± 20	145 ± 19	144 ± 21	0.16	3.3 (−5.3 to 12.0), 0.44
SBP (mmHg)	186 ± 32	181 ± 31	191 ± 34	0.31	−10.1 (−30.4 to 10.5), 0.31
DBP (mmHg)	92 ± 10	95 ± 8	89 ± 11	0.57	5.7 (−0.4 to 11.9), 0.07
Peak workload (W)	104 ± 36	92 ± 29	117 ± 40	0.70	−24.6 (−43.1 to −6.2), 0.01
RER	1.16 ± 0.1	1.17 ± 0.1	1.15 ± 0.1	0.10	0.01 (−0.05 to 0.08), 0.69
$\dot{V}$O_2_ absolute (L/min)	1.30 ± 0.3	1.19 ± 0.48	1.57 ± 0.51	0.34	−0.1 (−0.4 to 0.1), 0.12
$\dot{V}$O_2_ relative (L/kg/min)	15.0 ± 3.2	15.31 ± 5.0	17.6 ± 5.0	0.22	−1.3 (−3.7 to 0.9), 0.04
$\dot{V}$O_2_ (% predicted)	41 ± 5	42 ± 8	46 ± 5	0.58	−4.3 (−7.7 to 0.9), 0.01
O_2_ pulse (ml/beat)	9.7 ± 2.5	8.4 ± 1.8	9.3 ± 2.0	0.29	0.9 (−0.1 to 1.9), 0.04
O_2_ pulse (% predicted)	53 ± 6	51 ± 4	54 ± 3	0.20	3.2 (−7.1 to 13.6), 0.53
OUES L/min	1.8 ± 0.7	2.0 ± 0.3	2.3 ± 0.3	0.19	0.2 (−1.2 to 0.7), 0.56
OUES (% predicted)	56 ± 6	50 ± 4	62 ± 6	0.45	−12.0 (−25.9 to 1.8), 0.04

Notes: Student’s *t*-test; SBP: systolic blood pressure; DBP: diastolic blood pressure; HR: heart rate; WR: work rate; $\dot{V}$O_2_: oxygen uptake; RER: respiratory exchange ratio; VE: Minute ventilation; VCO_2_: carbon dioxide production; VE/VCO_2_ slope: linear relation between minute ventilation and carbon dioxide production; SBP: Systolic blood pressure, DBP: Diastolic blood pressure; OUES: oxygen uptake efficiency slope.

**Table 4:** Multiple linear regression analyses to evaluate the influence of variables on $\dot{V}$O_2_ relative peak (L/kg/min).

Variables	ß	CI 95%	*p* Value
Age	0.25	0.002 to 0.37	0.045
Hospital stays (days) (< and >10 days)	0.30	0.000 to 0.012	0.050
Diabetes	0.059	−0.17 to 0.27	0.67
FEV_1_/FVC (%)	0.297	0.042 to 2.82	0.044

Notes: Forced expiratory volume at the first minute (FEV_1_) and forced vital capacity (FVC).

## Data Availability

Dataset available on request from the authors.
